# Evaluation of citrus fiber as a natural alternative to sodium tripolyphosphate in marinated boneless broiler chicken breast and inside beef skirt (transversus abdominis)

**DOI:** 10.5713/ab.22.0145

**Published:** 2023-11-13

**Authors:** Kendal R. Howard, Cheyenne L. Runyan, Allen B. Poe, Andrew M. Cassens, Lea A. Kinman

**Affiliations:** 1 Department of Animal Science, Tarleton State University, Stephenville, TX 76401, USA; 2 Department of Agricultural Education and Communication, Tarleton State University, Stephenville, TX 76401, USA; 3 Department of Agriculture, Nutrition, and Human Ecology, Prairie View A&M University, Prairie View, TX 77446, USA

**Keywords:** Beef Skirt, Chicken Breast, Citrus Fiber, Sensory, Shear Force, Sodium Tripolyphosphate

## Abstract

**Objective:**

This research was conducted to evaluate the effects of citrus fiber (CF) as a natural alternative to sodium tripolyphosphate (STPP) in marinated broiler boneless chicken breast and inside beef skirt on overall retention rate, shear force, and consumer sensory attributes.

**Methods:**

Five different marinade formulations were targeted to include 0.9% salt, either 0.25% or 0.50% STPP or CF and water on a finished product basis. Water and salt only were considered the negative control (CON). Chicken breasts (n = 14) and inside beef skirt (n = 14) were randomly assigned to a treatment, raw weights recorded and then placed in a vacuum tumbler. Marinated weights were recorded, individually packed, and randomly assigned to either retail display for 10-day retention rate, shear force analysis, cook loss, or consumer sensory panel.

**Results:**

Pickup percentage, and overall retention was similar among treatments for chicken breast and inside beef skirt. Citrus fiber treatments resulted in higher cooking loss compared to the CON in chicken breast; though, CF050 resulted in similar cooking loss compared to STPP025 in inside beef skirt. No differences were found in sensory attributes for chicken breast, however, WBSF data showed CF025 was tougher than CF050, STPP050, and CON. Inside beef skirt with CF050 were least liked overall by the consumer panel.

**Conclusion:**

Citrus fiber included in marinades at a lower percentage rate can produce similar texture characteristics, and sensory properties compared with those marinated with STPP.

## INTRODUCTION

The growing skepticism from consumers regarding food additives has created a demand and market for “clean label” products. Awareness in clean label food products has increased due to consumer perception that natural ingredients are healthier and higher quality [1]. While all food additives provide specific functions, consumers perceive many as “unnatural,” “unhealthy,” or “unsafe.” Some ingredients that are criticized are sodium nitrate, sodium erythorbate, and phosphate. It had been established that cultured celery juice powder and cherry powder/acerola are acceptable natural alternatives for sodium nitrite and erythorbate in cured meat products [2]. Sodium tripolyphosphate (STPP) is a food additive used in meat products for its contribution to higher yields, improving sensory characteristics, specially texture. With the application of phosphates in marination products may experience to increase cook yield, water holding capacity, and juiciness. Complete elimination of phosphate is not plausible, so finding alternatives is at the forefront of the meat industry. One alternative that has shown potential is citrus fiber (CF). Citrus fiber (a by-product of the juicing industry) is produced from orange pulp, core, and peel. Due to its high surface area and fiber content, it functions to improve water retention, texture and gelation. This functionality shows potential to replace phosphate in marination formulations. Therefore, the objective of this study was to investigate the effect of CF use in marinated chicken breast and inside beef skirt on retention rates, cooking loss, and palatability traits.

## MATERIALS AND METHODS

### Marinade preparation

Marinades were prepared by adding the following ingredients to water: treatments; STPP (purchased from Innophos, Cranbury, NJ, USA) or CF (Citri-Fi 100; obtained from FiberStar, River Falls, WI, USA) when present, and salt, to make certain each ingredient was incorporated an emulsion blender was used until well blended. Marinade formulations were targeted to include 0.90% salt across all treatments, and either 0.25% or 0.50% STPP or CF and water on a finished product basis (Table 1).

### Marination and retention

Marination treatment and fourteen randomly assigned chicken breasts (raw weight recorded) were placed into a vacuum tumbler set at 15 pound per square inch (PSI) for 25 min operated at 45 rpm, following tumbling marinated weight was immediately recorded. Chicken breasts (n = 6/treatment) were vacuum packaged and placed in a retail display case to determine 10 d retention rate. Eight chicken breasts/treatment were frozen until Warner Bratzler Shear Force (WBSF) and consumer panel were performed. Inside beef skirt (n = 14/treatment) were randomly assigned to a marination treatment, placed into a vacuum tumbler set at 15 PSI for 15 min operated at 45 rpm. Raw weight was collected before placed in tumbler and marinated weight was collected after being placed on a rack to collect drip loss for 15 min and reweighed. Inside beef skirt (n = 6/treatment) were vacuum packaged and placed in a simulated retail display case to determine 10 d retention rate. Eight inside beef skirt were frozen until slice shear force (SSF) and consumer panels were performed. On day 10, meat samples were removed from bags and the bags were weighed to collect purge weight. Bags were then rinsed, and dried using a towel, then bags were weighed (net weight). Retention rate was calculated by dividing purge weight by the net weight and multiplying by 100.

### Cooking loss

Chicken breasts (n = 8/treatment) were thawed at 4°C for 24 h, and individual raw weight was recorded. Individual chicken breasts were butterflied to ensure even cooking, placed on a clamshell grill set at 177°C and removed when internal temperature reached 73°C. Cooked weights were immediately recorded to determine cook loss. Cook loss was calculated on a percentage basis by taking the total raw weight/treatment minus total cooked weight/treatment and dividing it by the total raw weight. Inside beef skirt (n = 6/treatment) were thawed at 4°C for 24 h, raw weight of individual skirt was recorded. Inside beef skirt were placed in an aluminum-lined baking pan, covered in foil, and cooked at 177°C in a Vulcan convection oven to an internal temperature of 71°C. Cooked weights were immediately recorded, and cook loss was calculated.

### Shear force analysis

Chicken breasts (n = 6/treatment) were thawed at 4°C for 24 h, placed in an aluminum-lined baking pan, covered in foil, and cooked at 177°C in a Vulcan convection oven to an internal temperature of 73°C, and were then chilled for 18 h at approximately 2°C to 4°C. Chilled chicken breasts were allowed to acclimate to room temperature for 30 min., then six 1.3-cm cores were removed parallel to the muscle fibers from individual chicken breasts using a hand-held coring device. Cores were sheared once, perpendicular to muscle fibers using the TA.XTPlus with a WBSF probe. The following settings were used on the TA.XTPlus; test speed = 2 mm/s, post-test speed = 10 mm/s, distance = 50 mm, and force = 5 g. Peak-shear force was recorded, and the mean-peak-shear-force values were used for statistical analysis. Inside beef skirt (n = 6/treatment) were cooked in the same manner to determine SSF. Two slices (1 cm×5 cm) were removed from each cooked inside skirt parallel to the longitudinal orientation of the muscle fiber using a 45° slice box and a double blade knife. Slices were sheared once, perpendicular to muscle fibers using the TA.XTPlus with a SSF probe. Settings for the TA.XTPlus were the same as WBSF.

### Consumer sensory panel

A consumer panel (n = 50) was conducted for sensory attribute evaluation. Chicken breasts (n = 8/treatment) were assigned a random number and thawed at 4°C for 24 h, butterflied, and cooked on a clamshell grill set at 177°C to an internal temperature of 73°C monitored using calibrated thermometers. Inside beef skirt (n = 8/treatment) were cooked on a clamshell grill set at 177°C to an internal temperature of 71°C. All cooked chicken breasts and inside beef skirt were cut into 1.37 cm cubes and 2 cubes from each treatment were placed into individually labeled sample cups with a lid and immediately served to panelists. Panelists were provided deionized water and unsalted saltine crackers to cleanse their palettes before and between samples. Consumer sensory panel methods were approved by the Institutional Review Board (1696660-1). Within the demographic ballot, panelists were asked to rank the following factors on intent to purchase from most to least important: color, price, labeling claims, ingredient statement, brand name. Panelists were then asked to evaluate chicken breast and inside beef skirt attributes based on a 9-point scale. Attributes included: overall liking (1, dislike extremely; 9, like extremely), color liking (1, dislike extremely; 9, like extremely), juiciness liking (1, dislike extremely; 9, like extremely), texture liking (1, dislike extremely; 9, like extremely) and likelihood to purchase on a 5-point scale (1, definitely would not buy; 5, definitely would buy).

### Statistical analysis

A completely randomized design was used to assign batch with treatments. Slice shear force and cooking loss data were analyzed using Statistix (Ver. 10.0 USA). Retention data were analyzed using the PROC GLM procedure of SAS v 9.4. For consumer panel, data were analyzed using the PROC GLIMMIX procedure in SAS v 9.4. Separation of means was performed by using least square differences in Saxton’s PDMIX800 macro. Alpha was set at 0.05.

## RESULTS AND DISCUSSION

### Retention rate

Water holding capacity (WHC) of meat is defined as the affinity of meat to retain its own or added water during processing and considered as one of the important measurements of quality attributes to determine the possibility of using the meat in manufacturing [3]. Dietary fibers have been described as “the remnants of plant cells resistant to digestion by human enzymes whose components are hemicellulose, cellulose, pectin, lignin, oligosaccharides, gums and waxes [4]. In a recent study, pectin and cellulose were reported to be the most predominant polysaccharides in CF [5]. Pectin’s inherent viscous properties contributes to CF functionality. No differences were found among treatments in pickup percentage and retention rate of chicken breast and inside beef skirt (Table 2). Insoluble fiber of citrus albedo was responsible for lipid-holding capacity, whereas, the soluble portion is responsible for WHC [6]. This is significant since CF (Citri-Fi 100M40; FiberStar, USA) utilized in this study is approximately 35% soluble fiber [7]. In a study, Casco et al [8] found similar (p>0.05) pickup percentage at 20 min and 24 h retention in boneless, skinless chicken breast marinated with SavorPhos, a proprietary blend labeled as citrus flour, all-natural flavorings, and less than 2% sodium carbonate. Water-binding ability increased with increased phosphate concentrations was documented to be related to changes in ionic strength or increased phosphate binding to proteins [9]. The results from this study found that replacement of STPP with CF did not affect on overall retention rate.

### Cook loss

Cooking loss occurs through the release of fat and moisture, and is related to the binding ability between meat protein, fat, and moisture [10]. Chicken breast marinated with CF025, CF050, and CON had the highest cooking loss percentage, when compared to other treatments. In beef skirt steaks, CF025 and CF050 had higher cooking loss compared to STPP050, however, CF050 was found to have similar cooking loss percentage to STPP025 (Table 3). Cooking loss percentage decreased with the addition of SavorPhos compared to a phosphate blend [8]. Natural fibers, such as those extracted from citrus albedo, have been used to increase cooking yields. This increase is attributed to the ability of citrus albedo to bind oil and water in restructured and emulsion meat products [11,12]. The improvement in cooking performance due to citrus albedo addition appears to be related with their fat and water holding capacity. Lemon albedo has a high water and oil holding capacity [13], due to their soluble components, mainly pectin, which may constitute up to 25% of the tissue [14]. Inclusion of CF050 can be a more practical replacement for STPP with regards to cooking loss in beef products.

### Warner Bratzler shear force and slice shear force

Tenderness of meat products, together with juiciness, flavor, and color are the main characteristics that influence a consumers’ overall judgement of quality [15]. The fiber in CF form a “gel-like” network when hydrated with water and it is this functionality that shows promising results to contribute to water retention and texture in processed meat [5]. Chicken breasts marinated with CF025 were found to have significantly higher WBSF values compared to CF050, STPP050, and CON. Beef skirt steaks marinated with CF025, CF050, and CON had significantly higher SSF values compared STPP025 and STPP050, thus, indicating a tougher product (Table 4). Cooking has a major influence on tenderness. Myofibrillar and connective tissue proteins undergo several temperature and time dependent structural changes during cooking, which can directly impact product yield, texture, and overall eating quality. Thus, cooking can cause either tenderization or toughening with the net effect being dependent on the inherent composition and characteristics of the muscles, the method of heating, and the time/temperature combination [16–20].

### Consumer sensory panel

**Figure 1:**
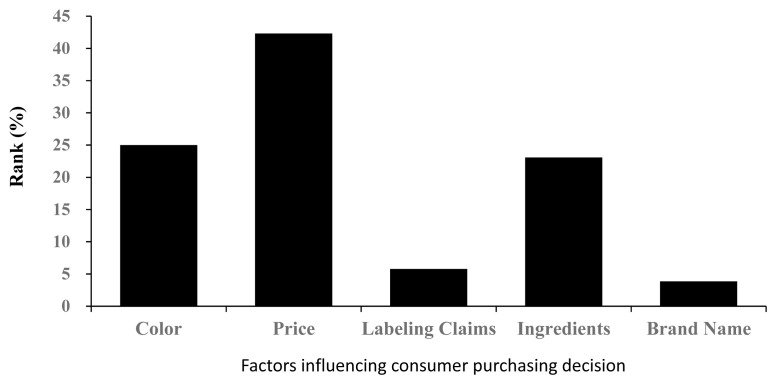
Distribution of factors that influence consumer purchasing decision (n = 50). Panelists were asked to rank each factor in order of preference when purchasing meat products. Price had the greatest influence on purchasing decisions.

The majority of participants (n = 50) ranked price as the leading purchasing decision factor (42.31%). Color (25.00%) and ingredients (23.08%) ranked second and third in this study (Figure 1). In a simulated shopping environment study for pork chops the majority of respondents reported using price to make their purchase decisions [21]. Color was reported to be one of the most important fresh meat characteristics at the point of purchase [22–24] because consumers use inadequate color as an indicator of spoilage and wholesomeness [25].

Consumer panelists found no differences among treatments for chicken breast palatability factors (Table 5). The results indicated that CF had no negative effect on sensory properties of marinated chicken breasts compared to STPP. Beneficial effects of marination on meat texture include juicier meat and reduction of water loss during cooking [26]. In a study [27] moistness in oven-roasted turkey breast with 0.50% CF was found to be significantly lower than control treatments. Consumer panelists also found no difference (p = 0.15) among treatments for color likeness in marinated inside beef skirt. However, panelists significantly preferred beef skirt marinated in STPP050 for juiciness, texture, and overall likeness, and would prefer to purchase compared to CF050 and CON (Table 6). Marination not only aids in improving tenderness but also WHC [28,29]. While tenderness has historically been identified as the most important palatability trait, overall beef eating quality is dependent upon three factors; tenderness, juiciness, and flavor, as well as the interaction among these traits [30–33]. Humans provide information on texture characteristics that instruments cannot such as mouthfeel, juiciness, tooth pack, moisture, and changes in texture while chewing [34]. Consumer satisfaction is crucial for repeat purchase of any product.

## CONCLUSION

Based on the current findings, CF has the potential to replace some of the functional properties of STPP in marinated chicken breasts and inside beef skirt. Citrus fiber as an alternative to STPP resulted in chicken with acceptable palatability attributes, as indicated by consumer preference. However, slight differences were found among inside beef skirt sensory, consumer panelist evaluations showed that CF050 received lower overall likeness. These results lead us to believe that CF has the potential to produce marinated chicken and beef with similar technological attributes, texture characteristics, and sensory properties as those made with STPP. Further studies should be conducted to examine the effects of CF on microbial inhibition during storage periods.

## Figures and Tables

**Table 1:** Marination formulations for chicken breast and inside beef skirt (percentage basis)

Items	CON	0.25CF	0.50CF	0.25STTP	0.50STTP
Chicken/beef	83.33	83.33	83.33	83.33	83.33
Water	15.76	15.51	15.26	15.51	15.26
Salt	0.90	0.90	0.90	0.90	0.90
CF	0.00	0.25	0.50	0.00	0.00
STTP	0.00	0.00	0.00	0.25	0.50

CON, control; CF, citrus fiber; STTP, sodium tripolyphosphate.

**Table 2:** Pickup percentage and retention rate of chicken breasts and beef skirt steaks

Treatment	Chicken		Beef	
	
	Pickup (%)	Retention (%)	Pickup (%)	Retention (%)
Control	15.87±2.46	95.71±1.19	45.15±6.85	96.38±1.32
0.25CF	13.12±2.31	96.08±1.19	48.64±1.32	94.85±1.32
0.50CF	10.39±2.35	95.56±1.19	40.98±6.24	97.47±1.32
0.25STPP	14.14±0.92	96.04±1.19	51.70±2.16	96.58±1.32
0.50STPP	14.55±1.54	96.53±1.19	47.29±11.60	95.55±1.32
p-value	0.22	0.48	0.78	0.14

CF, citrus fiber; STPP, sodium tripolyphosphate.

**Table 3:** Cooking loss for chicken breast and beef skirt steaks

Treatment	Cooking loss (%)	

	Chicken	Beef
CON	20.25^a^±2.43	34.09^a^±4.78
CF025	20.53^a^±2.95	27.53^a^±6.65
CF050	20.30^a^±1.91	24.49^ab^±0.97
STPP025	16.70^b^±2.27	12.83^bc^±6.29
STPP050	13.46^c^±3.20	9.93^c^±1.85

CF, citrus fiber; STPP, sodium tripolyphosphate.

a–c Means within a column without a common superscript are different.

**Table 4:** Warner Bratzler shear force (WBSF) values for chicken breast and slice shear force (SSF) values for inside beef skirt

Treatment	Chicken	Beef
	
	WBSF (g)	SSF (g)
CON	948.33^b^±515.98	7,637.40^a^±1,957.70
CF025	1,262.00^a^±577.81	8,931.40^a^±2,874.20
CF050	988.31^b^±279.86	9,021.20^a^±2,412.70
STPP025	1,075.80^ab^±420.04	4,740.00^b^±2,769.00
STPP050	976.15^b^±220.52	5,515.40^b^±1,566.90

CF, citrus fiber; STPP, sodium tripolyphosphate.

a,b Means within a column without a common superscript are different.

**Table 5:** Consumer panelist evaluation for marinated chicken breast palatability attributes^1)^ and likelihood to purchase^2)^

Treatment	Color	Texture	Juiciness	Overall	Likelihood to purchase
CON	6.92	6.82	6.92	6.43	3.41
CF025	7.10	6.70	6.28	6.42	3.35
CF050	6.59	6.62	6.48	6.21	3.31
STPP025	7.22	7.17	6.85	7.09	3.73
STPP050	6.79	6.39	6.75	6.37	3.45
Pooled SEM	0.09	0.11	0.10	0.10	0.01
p-values	0.25	0.26	0.29	0.09	0.24

CF, citrus fiber; STPP, sodium tripolyphosphate; SEM, standard error of the mean.

1) Consumer panelist scale: 1, extremely dislike; 9, extremely like.

2) Likelihood to purchase scale: 1, definitely would not buy; 5, definitely would buy.

**Table 6:** Consumer panelist evaluation for marinated inside beef skirt on palatability attributes^1)^ and likelihood to purchase^2)^

Treatment	Color	Texture	Juiciness	Overall	Likelihood to purchase
CON	5.73	5.03^c^	6.45^b^	5.48^b^	2.85^b^
CF025	6.51	5.93^ab^	6.81^ab^	5.90^ab^	3.10^ab^
CF050	5.98	5.07^bc^	6.23^b^	5.21^b^	2.68^b^
STPP025	5.75	5.57^bc^	7.40^a^	5.82^ab^	2.96^b^
STPP050	6.21	6.57^a^	7.34^a^	6.51^a^	3.42^a^
Pooled SEM	0.11	0.13	0.09	0.12	0.07

CF, citrus fiber; STPP, sodium tripolyphosphate; SEM, standard error of the mean.

1) Consumer panelist scale: 1, extremely dislike; 9, extremely like.

2) Likelihood to purchase scale: 1, definitely would not buy; 5, definitely would buy.

a–c Means within a column without a common superscript are different.
